# Developing A Minimum Data Set for A Rheumatoid Arthritis Registry in Iran

**DOI:** 10.31138/mjr.33.1.55

**Published:** 2022-03-31

**Authors:** Mostafa Langarizadeh, Nahid Mehrabi, Tania Azadi, Esmaeil Mehraeen, Arman Ahmadzadeh

**Affiliations:** 1 Department of Health Information Management, Iran University Of Medical Sciences, Tehran, Iran,; 2 Department of Health Information Technology, Army University Of Medical Sciences, Tehran, Iran,; 3 Tehran University of Medical Sciences, Tehran, Iran,; 4 Department of Health Information Technology, Khalkhal University of Medical Sciences, Khalkal, Iran,; 5 Department of Rheumatology, Shahid Beheshti University of Medical Sciences, Tehran, Iran

**Keywords:** minimum data set, rheumatoid arthritis, registry, healthcare information system, Iran

## Abstract

**Background:**

Using Minimum Data Set (MDS) is the first step in creating and developing a health care information system; it includes standard and key data elements to capture and manage patient care.

**Aims:**

This study aimed to develop an MDS in order for using it for designing registry of patients with rheumatoid arthritis in Iran.

**Methods:**

This study was conducted at two stages in 2018. In stage one, qualitative method and semi-structured interview were used to identify the registry data elements of patients with rheumatoid arthritis. Collected data was analysed using content analysis method. In stage two, using Delphi method, the developed data set was revised and validated by 15 rheumatologists. Descriptive statistics using SPSS software was used to analyse the data in Delphi.

**Results:**

The final MDS included 22 data elements, which were divided into two major categories of management data (including demographic data, and admission and discharge) and clinical data (including patient examination, treatment plans, and medication prescribed by physician).

**Conclusion:**

Minimum data set is one of the standard data collection tools playing an important role in health care data management. This study presented a MDS as a platform for creating a rheumatoid arthritis registry system in Iran recommended by rheumatologists.

## INTRODUCTION

Rheumatoid Arthritis (RA) is one of the most common chronic and incurable systemic autoimmune and inflammatory diseases, which leads to destruction, deformity, or reduced function of joints.^1^ The disease accompanies by pain, swelling, erosive damage, stiffness, and deformation of joints. The joint stiffness reduces the strength of muscles that are attached to affected joints and leads to movement disability.^2–4^ The prevalence rate of this disease is estimated to be one percent in the world; its incidence is usually prevalent in the third to fifth decades of life and is three times more common among women.^5^ Due to chronic nature of RA, it is necessary to acquire knowledge of this disease in order to make appropriate decisions to properly manage the health status of patients and create a treatment plan tailored to their lifestyle. The strategies to cope with such disease include suppressing inflammation and autoimmune responses, controlling pain, maintaining or improving joint movement and function, and increasing patients’ awareness of the disease process.^6,7^ In recent years, there have been significant developments in drugs and treatment strategies of RA.^8^

Developing a main resource for data collection in rheumatoid arthritis research:

1. Integrates data collection operations in future studies and observations.
2. Is as creating a common model for current databases.
3. Provides a template that can be used to collect data from routine clinical activities in a standard and appropriate way.^9^

The disease registry assists in evaluating and controlling rheumatology.^11^ In an organised disease registry system, the uniform data of a specific population with a specific disease or condition is collected and used for scientific, clinical, and health policy-making purposes. ^12^ This system has many benefits, such as improved patient care, reduced costs, and reduced medical errors.^13^

The Minimum Data Set (MDS) is essential for collecting high-quality data and establishing an integrated health information system and is the first step toward designing national registry systems.^14–17^

Considering the complications of RA, it is necessary to collect its data in a standard way at national level. This data may be used in the disease research. Thus, this study aimed to develop an MDS in order for clinical purposes and using it for designing a registry system of patients with RA in Iran to manage patients.

## MATERIALS AND METHODS

### Interview

This was a qualitative study through conducting interviews. The content analysis method was used to determine the MDS to be used in a RA registry system. The interviews were performed among 15 rheumatologists in five public hospitals (affiliated to medical universities in city of Tehran) during February 2018 to October 2018. The face-to-face semi-structured interview and focus group interviews were conducted for collecting the data. The snowball sampling technique was applied to identify the participants. The first interviewee was selected based on predefined criteria. The selection criteria were having at least a specialised doctorate degree and being a faculty member with at least 10 years of experience in the field of rheumatology, working in universities, hospitals, and rheumatology research centres. Data collection was carried out by means of a previously researcher-made interview guide, consisting of demographics data and semi-structure questions in the Persian language. Questions were asked about the major and standard data elements required for documenting RA patients’ care in Iran.

The interviews were recorded. The content analysis method was used to analyse the collected data, the main concepts and sub-topics were extracted from raw data, and were then coded. Then, the initial codes for the smallest meaning units were extracted from raw data. The initial codes were classified based on their similarities and differences; similar codes were placed under one class. All interviews were categorized into main topics and sub-topics. The results were classified and analysed manually without using software.

The validity of results (ie, coding) was evaluated by the participants. Also, the results were reviewed by the research team. The reliability of data was assessed by an individual who was expert in qualitative research. This study observed ethical principles such as privacy of information, obtaining informed consent for interview, and granting the right to withdraw from research at any stage.

### Delphi Process

This stage of the research was performed in response to the goal of validation of the proposed MDS of RA registration system for Iran. The research method at this stage was quantitative. The participants in round one and round two of Delphi were the same as the participants in the interview, and included 15 rheumatologists.

A structured questionnaire including closed-ended questions based on the consensus of experts’ opinions was used to collect data at this stage. An open-ended question was provided at the end of each section for any additional suggestions. In this questionnaire, the five-choice Likert scale was used based on the level of importance of each data element (Very Important, Important, Moderately Important, Slightly Important, Not Important). The content validity of the Delphi questionnaire was confirmed by three rheumatologists. Two rounds of Delphi were performed to reach final consensus. Frequencies, percentages, interquartile ranges and median scores were calculated to conclude the degree of agreement for each data element. Data elements were accepted if they attained more than 75% of collective consensus of (5=Very Important) and (4=Important). Collective consensus of items less than 50% and between 50% to 75% were removed and were sent for the next round respectively.

## RESULTS

The interviewees included 15 (5 female and 10 male) rheumatologists working in hospitals affiliated to medical universities in city of Tehran. Most of the interviewees were in the age group of 40–49 years old, had a subspecialty academic degree, and their work experience was between 5–20 years. The average time spent on each interview was 55 minutes.

### Identification of the MDS Data Elements

Qualitative content analysis of the interviews showed that the minimum data set should be in the form of clinical data, physician evaluation, patient evaluation, prescribed drugs and treatment recommendations.

*“The minimum clinical data set is a method of organizing information that is collected with the aim of determining information elements for each patient and provides a similar definition for common terms and information elements (I3)”.*

In the field of clinical data, determining the type of data is necessary to support medical care.

*“Capturing a wide range of clinical data is necessary to describe diagnoses, interventions, and treatment outcomes (I9)”.*

In this context, a variety of clinical data, including physician evaluations, patient assessments of the recovery process, treatment recommendations, and prescription drugs are noteworthy. Interviewees believed that:

*“Rheumatoid arthritis is known as a chronic inflammatory disease, so describing the stages of the disease based on the type of treatment and how the doctor diagnoses it according to the examinations and interventions performed at each visit must be captured in the registration system (I4)”.*

### Verification of the MDS Data Elements

**Figure 1.**
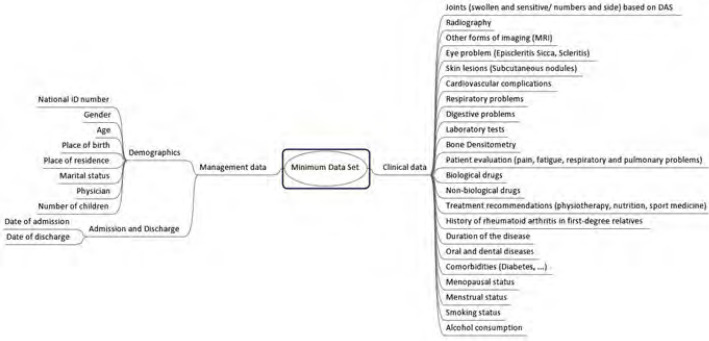
Final validated MDS for RA registry in Iran.

The MDS included two major categories of management data (including demographic data, and admission and discharge) and clinical data (including patient examination, treatment plans, and medication prescribed by physician). **Table 1** shows the frequency of answers regarding the importance of each of the MDS elements of RA registry in Iran in the first round of Delphi process. In the first round of Delphi, all variables were agreed on by the experts except for the hospital name (clinic), oral diseases, as well as comorbidities (such as diabetes, etc.) which were transferred to the second round of Delphi, due to lack of consensus. According to **Table 2**, in the second round of Delphi, the name of hospital/clinic and was removed due to lack of agreement. The oral and comorbidities such as diabetes were confirmed. In the second round of Delphi, the experts suggested that the data elements including menopause, menstruation, alcohol and smoking, and number of children to be added to MDS; all of these items were accepted and confirmed. The final MDS which was proposed and validated by the experts for using in RA registry in Iran is presented in **Figure 1**.

**Table 1.** Frequency of answers regarding the importance of each of MDS elements of RA registry in Iran in the first round of Delphi.

**Data element Very Important Important Frequency (Percentage) Frequency (Percentage)**			**Level of Importance**					**Median**	**Result**
			**Moderately Important**	**Slightly Important**	**Not Important**				
			**Frequency (Percentage)**	**Frequency (Percentage)**	**Frequency (Percentage)**				
**Managerial data**	**Demographic characteristics**	**National ID number**	13 (86.7)	2 (13.3)	0	0	0	5	Agreed
		**Gender**	15 (100)	0	0	0	0	5	Agreed
		**Age**	15 (100)	0	0	0	0	5	Agreed
		**Place of birth**	14 (93.3)	1 (6.7)	0	0	0	5	Agreed
		**Residence location**	12 (80)	1 (6.7)	2 (13.3)	0	0	5	Agreed
		**Marital status**	12 (80)	3 (20)	0	0	0	5	Agreed
		**Physician**	8 (53.3)	7 (46.7)	0	0	0	5	Agreed
		**Hospital / clinic name**	3 (20)	7 (46.7)	5 (33.3)	0	0	4	Second round
	**Admission and discharge**	**Date of admission**	5 (33.3)	10 (66.7)	0	0	0	4	Agreed
		**Date of discharge**	5 (33.3)	10 (66.7)	0	0	0	4	Agreed
	**Joints (swollen and tender/number and position) based on DAS**		12 (80)	0	0	3 (20)	0	5	Agreed
	**Radiography**		9 (60)	6 (40)	0	0	0	5	Agreed
**Clinical data**	**Other forms of imaging (MRI)**		10 (66.7)	5 (33.3)	0	0	0	5	Agreed
	**Eye problems (Episcleritis, Sicca, Scleritis)**		11 (73.3)	4 (26.7)	0	0	0	5	Agreed
	**Skin lesions (Subcutaneous nodule)**		11 (73.3)	4 (26.7)	0	0	0	5	Agreed
	**Cardiovascular complications**		11 (73.3)	4 (26.7)	0	0	0	5	Agreed
	**Respiratory problems**		11 (73.3)	4 (26.7)	0	0	0	5	Agreed
	**Digestive problems**		8 (53.3)	7 (46.7)	0	0	0	5	Agreed
	**Laboratory tests**		11 (73.3)	4 (26.7)	0	0	0	5	Agreed
	**Bone Densitometry**		12 (80)	3 (20)	0	0	0	5	Agreed
	**Patient evaluation (pain, fatigue, respiratory and pulmonary problems)**		15 (100)	0	0	0	0	5	Agreed
	**Biological drugs**		11 (73.3)	4 (26.7)	0	0	0	5	Agreed
	**Non-biological drugs**		11 (73.3)	4 (26.7)	0	0	0	5	Agreed
	**Therapeutic recommendations (physiotherapy, nutrition, sport medicine)**		5 (33.3)	10 (66.7)	0	0	0	4	Agreed
**History of RA in first-degree relatives**			11 (73.3)	4 (26.7)	0	0	0	5	Agreed
**Duration of the disease**			11 (73.3)	4 (26.7)	`	0	0	5	Agreed
**Oral and dental diseases**			5 (33.3)	6 (40.0)	4 (26.7)	0	0	4	Second round
**Comorbidities (diabetes, etc.)**			10 (66.7)	1 (6.7)	4 (26.7)	0	0	5	Second round

**Table 2.** Frequency of answers regarding the importance of each of MDS elements of RA registry in Iran in the second round of Delphi.

**Minimum data set**	**Level of Importance**					**Median**	**Result**
	**Very Important**	**Important**	**Moderately Important**	**Slightly Important**	**Not Important**		
	**Frequency (Percentage)**	**Frequency (Percentage)**	**Frequency (Percentage)**	**Frequency (Percentage)**	**Frequency (Percentage)**		
**Hospital / clinic name**	3 (20)	0	6 (40)	1 (6.7)	5 (33.3)	3	Removed
**Oral and dental diseases**	6 (33.3)	7 (46.7)	2 (13.3)	0	0	3	Agreed
**Comorbidities (Diabetes, etc.)**	6 (33.3)	7 (46.7)	2 (13.3)	0	0	3	Agreed
**Menopausal condition** ♦	9 (60)	6 (40)	0	0	0	5	Agreed
**Menstrual status** ♦	9 (60)	6 (40)	0	0	0	5	Agreed
**Alcohol consumption** ♦	9 (60)	6 (40)	0	0	0	5	Agreed
**Smoking** ♦	9 (60)	6 (40)	0	0	0	5	Agreed
**Number of children** ♦	9 (60)	6 (40)	0	0	0	5	Agreed

♦: Items that were suggested by the experts in the first round of Delphi to be added to MDS.

## DISCUSSION

This study showed that the MDS of registering patients with RA is divided into two major areas: management data and clinical data. As the end users of RA registry, the rheumatologists may play an effective role in its design, effectiveness, and efficiency.^18^ The MDS development process described here could prove beneficial for standardising data collection, managing patients’ care and improving quality of care. Different studies have pointed out the importance of standard clinical documentation^19^ including RA patients.^18,20–22^ Bajraktari studied 951 patients with RA and found a significant relationship between demographic characteristics data such as gender, nationality, marital status, education level, and occupation and prevalence rate of this disease.^21^ This study confirmed the necessity of registering demographic characteristics data including national code, age, gender, marital status, address, date of admission, date of discharge, too. In this study, RA registry is implemented in hospitals with RA patients; so “Date of admission” and “Date of discharge” was considered important and was confirmed to be added to the proposed MDS for the RA inpatients.

Moller-Bisgaard and Ostergaard (2014) showed that the optimal use of radiology information in registries and clinical trials may improve physicians’ knowledge of its benefits and improve clinical and laboratory procedures to control and predict the course of RA^23^; this is consistent with findings of the present study. The study on Lombardy Rheumatology Network (LORHEN) (2017) showed that the information of comorbidities such as diabetes in patients with RA is an essential element in registers^22^; this is consistent with findings of the present study.

In the second round of Delphi, this data element was confirmed by the experts as an essential element in RA registry. Early intervention and treatment are essential to prevent long-term complications. In this study, the rheumatologists stated that the biological and non-biological drugs are important; this is consistent with findings of Radner (2018).^9^ Lopez-Olivo (2015) studied the effect of drugs on patients with rheumatoid arthritis. Using clinical data available at rheumatoid arthritis registry system, his study showed that treating with combined drug is more effective than by a single drug.^24^ In our study, experts also, stated the importance of acquiring relevant data on treatment with drugs to effectively manage the course of the disease.

In the present study, the rheumatologists considered it necessary to conduct diagnostic tests to control the disease and evaluate the effectiveness of drugs. In this regard, Michaud (2016) studied the effectiveness of RA registry system in providing information. Examining five registers in which laboratory data of patients with RA was recorded, his study proposed value and importance of laboratory studies.^25^ In line with the findings of our study, experts stressed the need to capture laboratory tests data for an accurate diagnosis of the disease.

In this proposed MDS data needs to be collected whenever a patient visits a specialist, so a 24/7-based registry is recommended. This is in line with other recommendations.^19,29^ The several instruments for capturing data related to for instance, laboratory tests or drugs were not discussed in the focus groups and the Delphi process as the main aim of this study was first to identify required data items. However, as the standardisation of the clinical data which has to be collected by the MDS is of high importance, in further studies the best guideline and recommendations have to be identified for necessary clinical data elements.

In Iran as a LMIC, research, development and usage of the disease registries are under the influence of existing human and physical resources.^19,28^ So, it is applicable that a registry system serves for both clinical and administrative purposes. Although the purpose of the present study was determining the minimum data set for rheumatoid arthritis registry for clinical purposes, and to determine the incidence of the disease or other health outcomes, it is possible also to use the aggregated data to examine the trend of the disease over time, or to evaluate the provision of health services and identifying at-risk groups as well. It worth noting that the clinical purpose of this MDS were agreed upon with the physicians who took part in the study.

Collecting patient data in a standard way is essential to improve the quality and consistency of clinical services, facilitate collaborative and effective working, benchmark clinical services against quality indicators, and align treatment strategies and clinical research opportunities.^26,27^ In modern medicine, many data are generated; but there are always gaps between data collection to comprehension and interpretation.^28,29^ Moreover, the available data is in high volume and confusing. Therefore, the Minimum Data Set (MDS) is collected, which is the standard method for collecting key data elements to make them easy to understand and compare.^30^ This study enjoyed using the Delphi process to reach consensus and it could be considered a strength. Other studies such as the one by Nikiphorou et al. reached consensus by an opinion-based process.^31^

This study had some limitations. The participants in Delphi process were the same as the physicians who took part in the interviews and the focus groups. This could be probably the reason for high agreement in the Delphi process. Another limitation was that in the present study, clinical data capturing standards such as laboratory tests or drugs were not further discussed, as the main aim of this study was first to identify required data items for the clinical documentation of patients referring to RA clinics. This could be explored in future studies.

## CONCLUSION

In conclusion, making timely health care decisions is clinically and managerially dependent on accurate and well-organised data and information. Therefore, the data capture and data collection methods will affect the success of treatment of rheumatoid arthritis. Minimum data set is one of the standard data collection tools, which plays an important role in health care data management. The development of a verified list of minimum data items to support care management in RA provides a harmonised and standardised method in planning, quality improvement, reporting, and direct care delivery. The process followed in this study was largely successful, achieved participation of 15 rheumatologists, and could help the development of a verified list of minimum data items to track the clinical care and outcomes in patients with RA. The rheumatologists pointed out that capturing these elements as a well-coordinated data capture method is important and necessary in managing the course of disease in RA patients. Further future studies to implement the proposed MDS with national linkage is recommended to evaluate its efficiency. The MDS developed and confirmed in this study could be considered as an important way forward, optimising data for having an ultimate impact on patient care.
